# Optimizing nutrient utilization, hydraulic loading rate, and feed conversion ratios through freshwater IMTA-aquaponic and hydroponic systems as an environmentally sustainable aquaculture concept

**DOI:** 10.1038/s41598-024-63919-7

**Published:** 2024-06-27

**Authors:** Ashraf M. A.-S. Goda, Ahmed M. Aboseif, Mostafa K. S. Taha, Eman Y. Mohammady, Nevine M. Aboushabana, Hani M. Nazmi, Marwa M. Zaher, Hadir A. Aly, Mohamed A. S. El-Okaby, Nora Ibáñez Otazua, Mohamed Ashour

**Affiliations:** 1 National Institute of Oceanography and Fisheries, (NIOF), Cairo, Egypt; 2 INKOA SISTEMAS, S.L., Ribera de Axpe 11, Edificio D1, Dpto 208, 48950 Erandio, Spain

**Keywords:** Integrated multi-trophic system, Nutrient film technique, Floating Raft systems, Nitrogen, Phosphorous, Freshwater crayfish, Freshwater mussels, Zoology, Climate sciences, Environmental sciences

## Abstract

Water quality in land-based fish production can be controlled through either instantaneous water exchange or costly wastewater treatment followed by recirculation. Agricultural-aquaculture integration is an excellent alternative technique for reducing nutrient discharge levels, boosting profitability, and converting fish culture wastewater into valuable products. The current study employed a solar energy system to power two separate IMTA-aquaponics systems (Nutrient Film Technique, NFT, and Floating Raft Systems, FRS) for the cultivation of Nile tilapia, African catfish, thin-lipped grey mullet, freshwater crayfish, freshwater mussels, and a variety of vegetables. Tilapia and catfish were fed exclusively on diets under the IMTA system. All wastewater from tilapia and catfish ponds, both dissolved and solid, flows sequentially to ponds containing other cultivated species. The water then flows through the IMTA system's terminal point to the NFT and FRS systems before returning to the tilapia and catfish ponds, allowing complete control of the nutrient flow throughout this entire circular system. Two 147-day production cycles were concluded. The results from the second production cycle are reported. Total biomass gain for aquatic species in the IMTA system was 736.46 kg, compared to 145.49 kg in the tilapia and 271.01 kg in the catfish monoculture systems. The current IMTA system had a cumulative feed conversion ratio (FCR) of 0.90, while the FCRs for tilapia and catfish were 1.28 and 1.42, respectively. Nile tilapia and catfish consumed 571.90 kg of feed containing 25.70 kg of nitrogen (N) and 9.70 kg of phosphorus (P), reflecting, and gaining 11.41 and 3.93 kg of dietary N and P, representing 44.40 and 40.46% dietary N and P retention, respectively. In the IMTA system, the addition of mullet and prawn as detrivores aquatic animals improves dietary N and P utilization efficiency to 59.06 and 51.19%, respectively, while the addition of mussels as herbivore animals improves dietary N and P utilization efficiency to 65.61 and 54.67%, respectively. Finally, using FRS and NFT as hydroponic systems increased dietary N and P efficiency to 83.51% N and 96.82% P, respectively. This study shows that the IMTA-Aquaponic system, as a bio-integrated food production system, can convert the majority of fish-fed residues into valuable products suitable for desert, rural, and urban areas in impoverished and developing countries.

## Introduction

Aquaculture, the world's fastest-growing food-producing sector, produced 88 million tonnes, or accounting for over 49% of worldwide fish production in 2020 (178 million tons)^1,2^. Egypt is a major producer of aquaculture in the Middle East and North Africa (MENA) area. Egyptian fish farms accounted for 80.5% of all fish landings in 2020, with over 1592 thousand tones of finfish and prawns (95% from private farms and 5% from government farms) and a total market value of approximately $USD 7.2 billion (1 $USD = 47.6 Egyptian pounds) With a total production of 418,000 tons, the remaining 19.5% of fish landings are from the wild in the Red Sea, Mediterranean, Nile River, and inland lakes^3^. The river Nile provides nearly all of Egypt's water (97%). Egypt is currently below the water poverty threshold due to a shortage of water supplies, demonstrating how freshwater scarcity has become a global phenomenon. Due to a lack of natural resources, especially land and water, as well as climate change and a rise in the frequency of severe weather events, Egypt is mainly unable to meet the needs of a growing population with locally grown food. As a result, Egypt is becoming increasingly sensitive to global commodity market shocks, raising concerns about food insecurity and malnutrition^2^.

Aquaculture enterprises usually spend between 50 and 60% of their operating budget on feed, making it a substantial cost component^4,5^. Fish may use up to 35% of their nutritional components to grow while the rest is discharged into the aquatic environment. To address these challenges, agriculture-based sustainable fish farming has been proposed as a way to increase food production while conserving water, recycling nutrients, and converting waste (water) into high-value resources^6^. Compared to traditional farming, some integrated farms can reduce water consumption by 90%^7^.

Aquaculture in dry areas like Egypt must use as little fresh water as possible due to limited rainfall and adequate freshwater supplies^8^. Water quality in land-based fish production can be controlled through either a high rate of water exchange, which is costly, or by water treatment and subsequent recirculation, which is more expensive. To counterbalance high operating expenses, the aquaculture system's income must rise while operational costs are decreased. This is accomplished by using extra nutrients in aquaculture for fish, prawns, and shellfish to diversify the fish farm's production and improve the utilization of freshwater resources. Of course, this will help Egypt achieve its strategic goal of boosting aquaculture fish production to 2.5 million tons by 2030^9^.

Aquaponics is a soilless agriculture technique that supports aquaculture. Aquaponics originated thousands of years ago in China and Egypt when rice fields were combined with fish farming, such as carp and eels^10^. Water in an aquaponics system performs two functions: it habitats fish and grows vegetables, resulting in the simultaneous production of two products. The fish waste also fertilizes the water used to irrigate the plants, so cleaning the water for the fish. As a result, aquaponics, a bio-integrated food production system that can produce more food with less water, best practices to minimize agriculture's "water footprint" and manage natural resources smartly and efficiently, while also supplying people with the protein and minerals they require^11^.

Monoculture development is currently stalled because of increased input costs (including feed, power, and medications), environmental problems (including waste and poor water quality), and socioeconomic concerns (such as public opposition). Integrating extractive species (for example, invertebrates and/or seaweeds) into existing monoculture systems, such as integrated multi-trophic aquaculture (IMTA), has the potential to improve farm profitability, animal welfare, and economic benefits^12^. As a production system, IMTA-aquaponics may provide new insights into water management, increased fish biomass, improved feed conversion ratios, potential wastewater reduction, and recognition of external influences affecting monoculture production, as well as strategies to avoid them associated with diverse aquaponics crop production^13^.

For plants, the conductivity factor (CF) is important since a strong solution may burn the roots and create reverse osmosis^14^. Aquaponics systems have a higher CF when nutrients (mineral salts) dissolve in water. The CF value drops as minerals are absorbed by growing plants, indicating that more minerals are required by the plants. On a hot day, for example, if the plants are merely taking water from the system, we only need to supply water as the CF reading rises. Reverse osmosis, on the other hand, occurs when minerals are taken out of the plant because the solution on the outside of the plant is stronger than the solution inside, resulting in plant death^15^. Plant conductivity factor (CF) levels vary widely across crops, even during distinct growth stages. According to^16^, CF levels are normally highest in the mild winter months (cold, low light conditions) and lowest during the hot summer months (hot, high light conditions).

The purpose of the present study was to evaluate the feasibility of combining integrated multi-trophic aquaculture (IMTA), which produced Nile tilapia, *Oreochromis niloticus*, African catfish, *Clarias gariepinus*, Thin Lipped Grey Mullet, *Liza ramada*, freshwater prawn, *Macrobrachium rosenbergii*, and Freshwater mussels from family Iridinidae (*Aspatharia chaiziana* and *A. marnoi*), with hydroponic horticultural production (red and green leaf lettuce, chili and red peppers, head lettuce, cucumber, eggplant, tomato, and broccoli) using two hydroponic systems (i) Nutrient Film Technique (NFT) and ii) Floating Raft System (FRS) to maximize nutrient cycling resulting from aquatic animal and plant cultivation.

## Material and methods

### Experimental systems

**Figure 1:**
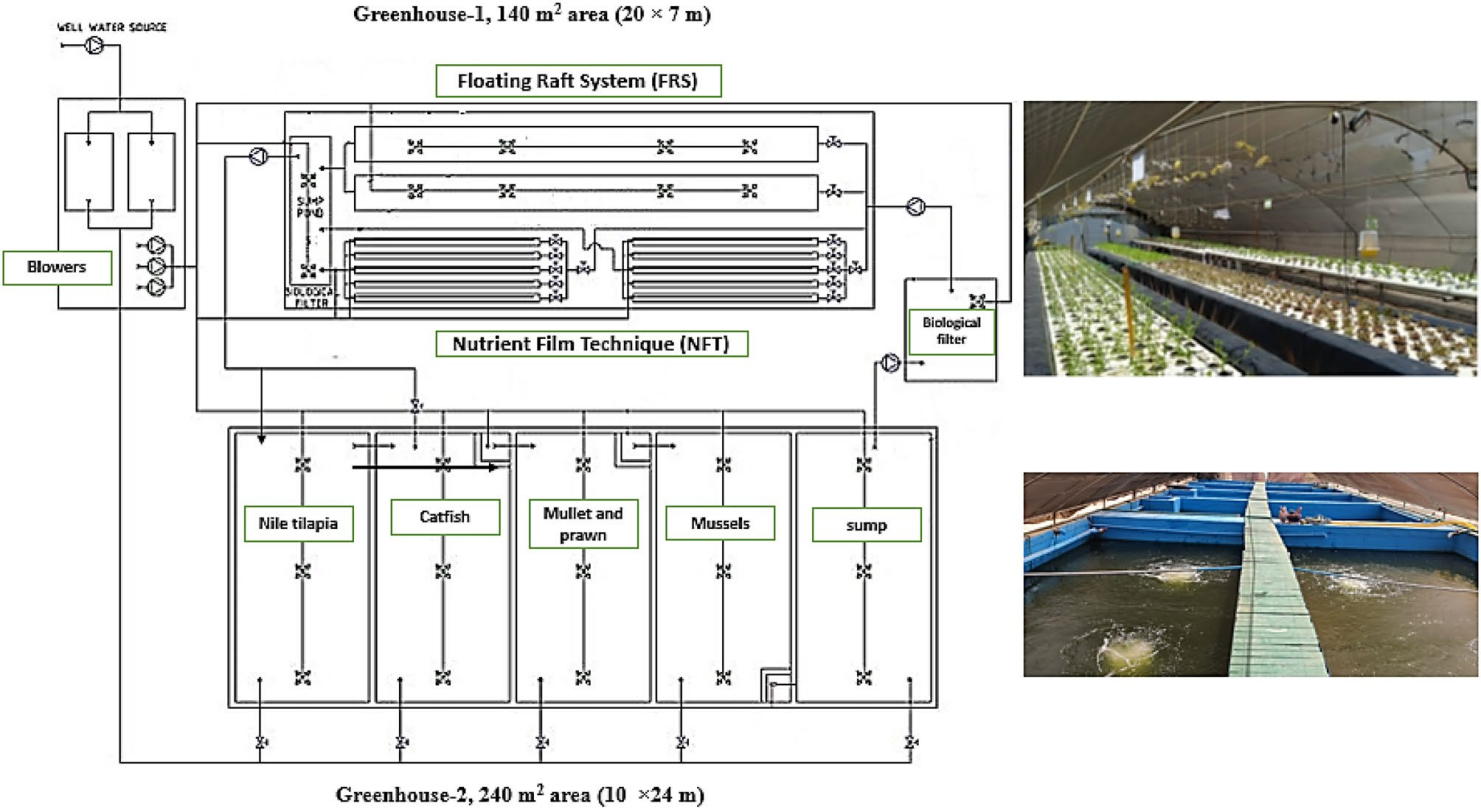
The schematic diagram explains the project system components.

The study was conducted at El-Kanater El-Khayria fish station, National Institute of Oceanography and Fisheries (NIOF), Kalubiya, Governorate, Egypt. During the experimental period, two low-tech greenhouses with simple metal structures comprise the testing area: Greenhouse-1, 140 m^2^ (20 × 7 m) with two NFT hydroponic units (122 m each) and two FRS hydroponic systems (20 m^3^ each); and Greenhouse-2, 240 m^2^ area (10 × 24 m) with 5 concrete ponds of 40 m^3^ for IMTA system production. In this experiment, the common and scientific names of fish species were updated using the Fishbase website (https://www.fishbase.se/search.php). Individual aquatic animals are cultured in an aquatic modular system, which allows discharged nutrients to be converted into valuable products. Figure 1 illustrates a diagram of the planned IMTA-aquaponics systems (IMTA-NFT and IMTA-FRS).

### Install the system and control the water flow

A self-priming pump with a flow rate of 16 m^3^ h^−1^ pumps fresh water from the well water source. The water then flows through Greenhouse 2 to fill the Nile tilapia and catfish ponds (first and second ponds), followed by a network of pipelines and tanks to the *L. ramada* and *M. rosenbergii* pond (third pond), and finally to the freshwater mussels (fourth pond). The sedimentation pond (fifth pond) serves as a mechanical filter, capturing a significant amount of solid waste. The sedimentation pond water was first filtered via a sand filter to remove leftover particles and organic material before passing through a large biological filter (6 m^3^), where ammonia is oxidized to nitrite and subsequently nitrate. The water flow within the system is controlled by three water pumps and manual ball valves.

After the nitrification process is completed, the water is filtered and carried to greenhouse-1 via the IMTA-FRS and IMTA-NFT (Fig. 1). The FRS and NFT units have a bulk water flow of about 4.8 m day^−1^ and 6.4 m day^−1^, respectively. The water terminates in a 3 m^3^ sump pond and then flows by gravity to Greenhouse-2 to tilapia and catfish ponds, with complete nutrient management over the entire circular system.

To match the size of the net pots, the pipes were drilled with holes 4 to 5 cm in diameter for the NFT. Plastic cups were used as planting containers with the bottom perforated (30 mm) to allow water to reach the roots of the plants. A closed aeration network distributes air to the system's various units via air nozzles. The aeration network is powered by four air blowers (Siemens, Munich, Germany, and SCHMALZ, Glatten, Germany) that operate at 30-min intervals using automatic timers 24 h a day. The system is powered by a mix of grid and solar energy. The solar panels (with a total area of 4 m^2^ and a capacity of 3 kW) produce enough energy to run the water pumps and air blowers from 8:00 to 16:00 h every day.

### Calculate the water flow rate (Qw) and the hydraulic loading rate (HLR)

The influence of water flow rate (Qw) and hydraulic loading rate (HLR) on water quality variables is determined by the following equation^17^:

1 $$ {\text{HLR }} = {\text{ Qw}}/{\text{entire}}\;{\text{trough}}\;{\text{surface}}\;{\text{area}} $$

The water flow rate (Qw) in a backyard-sized aquaponic system was calculated so that the volume of water equaled the fish tank passing through the biofilter twice per hour. The current design provides a water flow rate of 16 m^3^ h^−1^ (6 h).

### Water sample collection and analysis

Throughout the experimental period, water quality samples were analyzed using the Professional Plus (Pro Plus) Multiparameter Instrument (YSI Company, Yellow Springs Instrument, Yellow Springs, Ohio, USA), including temperature, electrical conductivity (EC), pH, dissolved oxygen (DO), and total alkalinity (TA) over the entire experimental period. The APHA^18^ standard procedures were used to determine the weak values of biological oxygen demand (BOD), chemical oxygen demand (COD), ammonia (NH^4^), nitrite (NO^2^), nitrate (NO^4^), orthophosphate (PO^4^), and total phosphorus (TP).

### Experimental aquatic organisms and culture techniques

All animal techniques in the present investigation were carried out following the relevant guidelines and regulations of the National Institute of Oceanography and Fisheries (NIOF). Tilapia fingerlings with an initial body weight of 25.4 ± 1.7 g were stocked in the first cement pond at a density of (15 fish m^−3^). Catfish weighing 173.27 ± 5.64 g were stocked in the second pond (40 m^3^) at a rate of 10 fish m^−3^. Water from the tilapia and catfish ponds is pumped separately to the third pond, which includes a polyculture of two benthic aquatic detritus species (*L. ramada* fry with an initial body weight of 0.2 ± 0.04 g and *M. rosenbergii* post-larvae with an initial body weight of 0.28 ± 0.2 g) at stocking density of (50 fry m^−3^ and 84 prawn m^−2^, respectively). The water was then delivered to the fourth cement pond stocked with freshwater mussels as filter-feeder species with an initial body weight of 138.58 ± 15.22 g obtained from wild sources (El-Kanater El-Khayria, El-Menoufy Branch of the River Nile) at stocking density of 2.5 kg m^−2^. All aquatic animals were acclimated to the experimental conditions for two weeks prior to the start of the study. During this time, Nile tilapia and catfish were only fed an experimental diet (30.58% and 19.38 MJ gross energy/kg, respectively). A tiny seine net was used to collect a sample of cultured fish, prawns, and mussels to measure weight increment and gain. During several of these weightings, sub-samples were collected for proximate body composition analysis. The initial sampling was done on the 30th day following stocking and every 15-day interval thereafter. The quantity of fish consumed daily was adjusted accordingly.

### Type of hydroponic systems

Two separate IMTA–Aquaponics (IMTA-NFT and IMTA-FRS) were evaluated. The slope of the channel, length, and rate of water flow were all considered in NFT to ensure that the plants received adequate water, oxygen, and nutrients^19^. The IMTA-NFT system is based on the idea that a shallow flow of constantly flowing water provides a continuous supply of water, nutrients, and oxygen to the bottom of the thick layer of roots that develops in the trough, while the top of the root mass is exposed to the air, thereby receiving an adequate oxygen supply.

The experimental FRS was a 20 m^2^ growing bed with dimensions of 20 × 1 × 0.3 m (L × W × D) and a black plastic liner (1 mm thickness). A 5 cm drain was established at the bottom of each bed's west side. A 5 cm thick hydroponic Styrofoam board cut to the size of the hydroponic bed was used to float the various plant heads, allowing the roots to remain suspended in the water. The IMTA-FRS principle states that plant roots grow directly into a container of water. The rafts provide maximum root exposure to nutrient-rich water. The Styrofoam boards also protect the water from direct sunlight, which helps to keep the water temperature low, which is beneficial for the fish. In both IMTA-NFT and IMTA-FRS systems, water is pumped from the tilapia (40 m^3^) and catfish (40 m^3^) ponds to the mullet and freshwater prawn pond (40 m^3^), then to the mussels pond (40 m^3^) then to the biological filter then to the hydroponic system finally recycled from the endpoint of either NFT or FRS system to both Nile tilapia and catfish ponds. Water was added to each aquaponics treatment system to compensate for evaporate-transpiration water losses. The weekly water loss is projected to be approximately 5% of the system volume. Every 30–45 min, the entire water cycle (fish-plants-fish) was evaluated to verify that fish nutrients were evenly distributed to plants. Each aquaponics system had one 20-m-long diffuser line installed at the bottom. An identical procedure was followed in each fish pond.

### Plants cultivate techniques

The source of plant seedlings used in the study was purchased from the Egyptian Ministry of Agriculture stores, Cairo. The plant collection and use were following all the relevant guidelines of the Egyptian Ministry of Agriculture. In FRS, plants were grown in Styrofoam rafts floating in the plant troughs, each of which contained 20 rafts covering a total area of 20 m^2^. Each raft (1.00 m^2^) was fitted with 25 holes (2″). The plants were cultivated in a greenhouse with natural photosynthesis, available radiation, and photoperiod at an air temperature of 25–32 °C. Each crop was rotated to reduce mineral concentration spikes caused by excessive fish waste consumption. Seedlings were planted on Styrofoam plates at a density of 72 plants/plat for cucumber, broccoli, and eggplant, and 96 plants/plat for bell and cayenne pepper, lettuce, and tomato, according to^20^. Each seedling was planted in plastic pots and then placed inside the holes in NFT and the floating Styrofoam plate in FRS. To protect each seedling, 3 cm square pieces of synthetic sponge were used to fit tightly inside the pots. Depending on the plant species, the plants were grown in the hydroponic systems from 33–45 to 80–90 days until the first harvest. The number of plants used in the trials is determined by plant species and life characteristics. Six vegetables were selected for a comparison study based on their economic value, suitability for cultivation, relative growth rates, and conductivity factor (CF, 100 μS = 1 CF). Three vegetable groups were examined based on their conductivity factor: Group 1, includes vegetables with low and intermediate CF (10–15 μS) like broccoli, lettuce, and cucumber, whereas Group 2 comprises vegetables with high CF (20–50 μS) like tomatoes, eggplant, and peppers. The date and weight of each plant were recorded for each harvest. Crop production, mineral concentrations in recirculation water, and ripe plant fruits were the primary focus of interest. The total biomass of growing plants for all plant crops was calculated at the end of each growth phase and adjusted based on trends in dissolved nutrient concentrations in recirculation water.

The following equations were used to calculate the average crop yield each harvest and the average yield growth per day:

2 $$ {\text{The}}\;{\text{average}}\;{\text{yield}}\;{\text{per}}\;{\text{harvest}} = \frac{{{\text{The}}\;{\text{total}}\;{\text{weight}}\;{\text{harvest}}}}{{{\text{The}}\;{\text{number}}\;{\text{of}}\;{\text{harvest}}}} $$

3 $$ {\text{The}}\;{\text{average}}\;{\text{yield}}\;{\text{growth}}\;{\text{per}}\;{\text{day}} = \frac{{{\text{The}}\;{\text{average}}\;{\text{yield}}\;{\text{per}}\;{\text{harvest}}}}{{{\text{The}}\;{\text{average}}\;{\text{number}}\;{\text{of}}\;{\text{days}}\;{\text{between}}\;{\text{harvests}}}} $$

### Aquatic organism growth and feed utilization indicators

The data include aquatic organism culture information such as stocking rates, stocking densities, growth, and feed utilization characteristics that describe the performance of fish, prawns, and mussels were calculated according to Goda et al.^21^ using the following equations:

4 $$ {\text{Weight}}\;{\text{gain }}\left( {{\text{WG}}} \right) \, = {\text{ Final}}\;{\text{body}}\;{\text{weight }}\left( {{\text{FBW}}} \right) \, - {\text{ Initial}}\;{\text{body}}\;{\text{weight }}\left( {{\text{IBW}}} \right) $$

5 $$ {\text{Specific}}\;{\text{growth}}\,{\text{rate }}({\text{SGR}}, \, \% /{\text{day}}) \, = \, \left[ {\left( {{\text{ln}}\;{\text{FBW }} - {\text{ ln}}\;{\text{IBW}}} \right)/{\text{t }} \times {1}00} \right] $$

where: FBW and IBW are final and initial body weight (g), respectively; ln = natural logarithmic; t = time in days

6 $$ {\text{Feed}}\;{\text{conversion}}\;{\text{ratio }}\left( {{\text{FCR}}} \right) \, = {\text{ Feed}}\;{\text{ intake }}\left( {\text{g}} \right)/{\text{weight}}\;{\text{gain }}\left( {\text{g}} \right) $$

7 $$ {\text{Protein}}\;{\text{efficiency}}\;{\text{ratio }}\left( {{\text{PER}}} \right) \, = {\text{ weight}}\;{\text{gain }}\left( {\text{g}} \right)/{\text{protein}}\;{\text{intake }}\left( {\text{g}} \right) $$

8 $$ {\text{Protein}}\;{\text{productive value}}\;\left( {{\text{PPV }}\% } \right) \, = \, \left( {{\text{protein}}\;{\text{gain }}\left( {\text{g}} \right)/{\text{protein}}\;{\text{ intake }}\left( {\text{g}} \right)} \right) \, \times { 1}00 $$

9 $$ {\text{Energy}}\;{\text{ retention }}\left( {{\text{ER }}\% } \right) \, = \, \left( {{\text{energy }}\;{\text{gain }}\left( {{\text{kJ}}} \right)/{\text{energy}}\;{\text{ intake }}\left( {{\text{kJ}}} \right)} \right) \, \times {1}00 $$

### Nitrogen (N) and phosphorus (P) calculation parameters

The mass balance was used to determine the N and P balances for fish-culture ponds and other experimental NFT and FRS systems using the following basic equations^22^:

10 $$ \begin{aligned} {\text{Nutrients }}\left( {\text{N or P}} \right){\text{ discharge }}\left( {\text{g}} \right) \, & = {\text{ Dietary }}\;{\text{nutrients }}\left( {{\text{N }}\;{\text{or }}\;{\text{P}}} \right){\text{ content }}\left( {\text{g}} \right) \,\\ &\quad - {\text{ fish}}\;{\text{ body }}\;{\text{deposition }}\;{\text{nutrients }}\left( {\text{N or P}} \right) \, \left( {\text{g}} \right) \end{aligned} $$

11 $$ {\text{Total }}\;{\text{Nutrient}}\;{\text{ gain }}\left( {\text{g}} \right) \, = {\text{ Tissue }}\;{\text{Nutrient }}\;{\text{gain }}\left( {\text{g}} \right) \, \times {\text{ total }}\;{\text{biomass}}\;{\text{ production }}\left( {{\text{g }}\;{\text{wet }}\;{\text{weight}}} \right) $$

12 $$ {\text{Nutrient }}\;{\text{intake }}\left( {\text{g}} \right) \, = {\text{ Total}}\;{\text{ feed }}\;{\text{intake }}\left( {{\text{g }}\;{\text{dry }}\;{\text{weight}}} \right) \times {\text{Nutrient }}\;{\text{feed }}\;{\text{content }}\left( \% \right) $$

13 $$ \begin{aligned} {\text{Total }}\;{\text{Nutrient }}\;{\text{gain}}\;{\text{ fish }}\left( {\text{g}} \right) \, & = \, \left( {{\text{Final }}\;{\text{bodyweight}}\left( {\text{g}} \right) \times {\text{final }}\;{\text{tissue}}\;{\text{ Nutrient }}\;{\text{content }}\left( \% \right)} \right) \,\\ &\quad {-} \, \left( {{\text{Initial }}\;{\text{bodyweight}}\left( {\text{g}} \right) \times {\text{initial }}\;{\text{tissue}}\;{\text{ Nutrient }}\;{\text{content }}\left( \% \right)} \right) \end{aligned} $$

14 $$ {\text{Nutrient }}\;{\text{discharge }}\left( {\text{g}} \right) \, = {\text{ Nutrient }}\;{\text{intake }}\left( {\text{g}} \right) \, {-}{\text{ Nutrient }}\;{\text{fish }}\;{\text{gain }}\left( {{\text{g }}\;{\text{wet }}\;{\text{weight}}} \right). $$

For mullets, prawns, and mussels, the following equations were used to predict (estimated) N and P balance values:

15 $$ \begin{aligned} &  {\text{Nutrient}}\left( {{\text{N }}\;{\text{or}}\;{\text{ P}}} \right){\text{gain}}\;{\text{ biomass }}\left( {\text{g}} \right) \, \left( {{\text{mullets}}, \, \;{\text{prawn}}, \, \;{\text{mussels}}} \right) \, \\ &\quad= \left( {{\text{Final}}\;{\text{ bodyweight}}\left( {\text{g}} \right) \times {\text{final }}\;{\text{tissue }}\;{\text{Nutrient }}\;{\text{content }}\left( \% \right)} \right) \, \\ &\quad\quad{-} \, \left( {{\text{Initial}}\;{\text{ bodyweight}}\left( {\text{g}} \right) \times {\text{initial}}\;{\text{ tissue }}\;{\text{Nutrient }}\;{\text{content }}\left( \% \right)} \right) \end{aligned} $$

16 $$ \begin{aligned} &  {\text{Nutrient}}\;{\text{discharge }}\left( {\text{g}} \right) \, \left( {{\text{mullets }}\;{\text{and }}\;{\text{prawn}}} \right) \, \\ &\quad= {\text{Nutrient}}\;{\text{discharge }}\;{\text{form }}\;{\text{Nile}}\;{\text{ tilapia }}\;{\text{pond }}\left( {\text{g}} \right) \, + {\text{Nutrient}}\;{\text{discharge}}\;{\text{ form}}\;{\text{ catfish}}\;{\text{ pond }}\left( {\text{g}} \right)) \, \\ & \qquad - {\text{Nutrient}}\left( {{\text{N }}\;{\text{or }}\;{\text{P}}} \right){\text{gain}}\;{\text{ biomass }}\;{\text{for}}\;{\text{ mullets }}\;{\text{and }}\;{\text{prawn }}\;{\text{pond }}\left( {\text{g}} \right) \end{aligned} $$

17 $$ \begin{aligned}  & {\text{Nutrient}}\;{\text{discharge }}\;{\text{from}}\;{\text{ mussels }}\left( {\text{g}} \right) \, \\ &\quad= {\text{Nutrient}}\;{\text{discharge}}\;{\text{ from }}\;{\text{mullets }}\;{\text{and}}\;{\text{ prawn }}\;{\text{pond }}\left( {\text{g}} \right) \, - {\text{Nutrient}}\left( {{\text{N}}\;{\text{ or}}\;{\text{ P}}} \right){\text{gain}}\;{\text{ biomass }}\;{\text{for }}\;{\text{mussels }}\left( {\text{g}} \right) \end{aligned} $$

### Proximate analysis

A random pooled sample of three healthy individual groups (5 individuals each) of each experimental aquatic species was selected, weighed, slaughtered, and immediately frozen at − 20 °C to determine the initial proximate body composition. Following the feeding trial, the same three individual groups of each experimental aquatic species were randomly selected from each experimental pond to determine the final proximate body composition. The proximate compositions of diets and aquatic animals' whole bodies were determined according to the procedures of Brett^23^ and AOAC^24^. The APHA^18^ procedure was used to determine the nitrate, ammonia, nitrite, total phosphorus (TP), and available phosphorus (PO_`_).

### Statistical analysis

The data was analyzed using the analysis of variance (ANOVA). Shapiro–Wilk and Bartlett's tests were used to confirm the normal distribution of the experiments. A Student's t-test was used to detect significant differences between treatments in aquatic animal growth, feed utilization, and production, whereas a one-way ANOVA was used to determine significant differences in plant production variables and nutrient removal among treatments. When significant F values were observed, Duncan's multiple range test was applied at the *p* ≤ 0.05 level to compare differences between treatment means^25^. All statistical tests were performed at a 5% level of significance^26^. SPSS software^27^ was employed (SPSS version 17.0, SPSS, Michigan Avenue, Chicago, IL, USA).

### Ethical approval

In accordance with ARRIVE guidelines, all experiments in this work were approved by the National Institute of Oceanography and Fisheries (NIOF) Committee for Institutional Care of Aquatic Organisms and Experimental Animals (NIOF- IACUC, Code: NIOF-AQ4-F-23-R-040).

## Results

All of the data that we will track are related to the second production cycle vs the first cycle, because the second cycle was more optimal in terms of implementation, and the challenges that we encountered in the first cycle were avoided.

### Hydraulic loading rate (HLR) and water flow rate (Qw)

Table 1 shows the temperature, pH, dissolved oxygen (DO), biochemical oxygen demand (BOD), chemical oxygen demand (COD), electrical conductivity (EC), total phosphorus (TP), and available phosphorus (PO_4_) levels in IMTA-FRS and IMTA-NFT systems. The water temperature, pH, and DO levels in the aquatic animal ponds ranged from 24.7 to 29.1 °C, 7.28 to 8.44, and 5.01 to 6.23 mg L^−1^, respectively. Table 1 illustrates how hydraulic loading rate (HLR) and water flow rate (Q) influence water quality variables (inflow and outflow) in IMTA, NFT, and FR systems. Increased water flow rates enhance NH_3_-N, NO_2_-N, NO_3_-N, and TP removal in NFT and FRS. In contrast to IMTA-NFT, all experimental plants in IMTA-FRS grew successfully in the hydroponic trough with no nutritional deficits or mineral imbalances. Plant production increased as the hydraulic loading rate increased from 4.8 m day^−1^ for FRS to 6.4 m day^−1^ for IMTA-NFT, leading to an increase in red, green, and head lettuce production (Table 2).

**Table 1:** Effect of hydraulic loading rate and water flow rate on the different water quality variables (Inflow and outflow) of IMTA-NFT and IMTA-FR systems in both first and second cycles.

Water quality variables*	Experimental treatments					
	IMTA		IMTA-FRS		IMTA-NFT	
Total area (m^3^)	40		20		122^#^	
Qw (m^3^ day^−1^)	96		96		96	
HLR (m day^−1^)	2.4		4.8		6.4	

	First cycle	Second cycle	First cycle	Second cycle	First cycle	Second cycle
EC (mS/cm)						
Inflow	703.38 ± 12.53	700.21 ± 10.76	706.75 ± 13.88	701.27 ± 12.11	714.63 ± 11.52	704.63 ± 14.55
Outflow	706.63 ± 13.12	710.16 ± 11.42	703.25 ± 7.34	708.17 ± 5.67	704 ± 12.25	710.34 ± 11.45
pH						
Inflow	7.24 ± 0.11	7.28 ± 1.41	7.37 ± 1.18	7.41 ± 0.14	8.4 ± 0.33	8.44 ± 0.19
Outflow	7.33 ± 0.42	7.37 ± 0.18	7.4 ± 4.23	7.44 ± 0.54	8.47 ± 1.11	8.51 ± 0.65
DO (mg/l)						
Inflow	5.17 ± 1.42	5.01 ± 1.21	6.11 ± 1.34	6.03 ± 1.09	6.31 ± 1.22	6.23 ± 1.01
Outflow	6.32 ± 1.33	5.12 ± 1.03	6.44 ± 1.83	5.99 ± 1.12	7.25 ± 1.00	6.47 ± 0.90
BOD (mg/l)						
Inflow	4.64 ± 1.25	5.01 ± 1.35	3.94 ± 1.05	3.96 ± 1.37	4.52 ± 1.65	4.49 ± 0.89
Outflow	4.41 ± 1.44	4.39 ± 1.21	3.9 ± 1.34	3.88 ± 1.09	4.67 ± 1.32	4.71 ± 0.91
COD (mg/l)						
Inflow	6.95 ± 1.34	6.92 ± 1.58	7.01 ± 1.66	7.04 ± 1.22	7.25 ± 1.12	7.22 ± 1.55
Outflow	7.18 ± 0.95	7.15 ± 1.54	6.95 ± 0.90	6.99 ± 0.57	6.81 ± 1.92	6.78 ± 0.87
TA (mg/l)						
Inflow	277.25 ± 3.87	276.97 ± 2.23	282.94 ± 1.65	281.1 ± 2.54	274.63 ± 3.12	273.09 ± 2.75
Outflow	280.44 ± 2.21	278.93 ± 2.67	281.63 ± 3.12	284.65 ± 4.73	273.44 ± 3.56	271.94 ± 5.12
TSS (mg/l)						
Inflow	23.73 ± 1.46	23.60 ± 1.56	14.18 ± 1.12	14.12 ± 2.23	15.1 ± 1.76	15.05 ± 3.42
Outflow	27.74 ± 1.23	27.31 ± 1.54	22.94 ± 2.08	22.58 ± 1.56	13.92 ± 1.89	13.7 ± 1.22
NH_3_ (µg/l)						
Inflow	627.78 ± 5.23	618.36 ± 4.45	322.49 ± 6.44	317.65 ± 4.73	304.38 ± 6.12	299.81 ± 8.54
Outflow	559.09 ± 9.42	552.38 ± 8.38	298.19 ± 7.11	294.91 ± 5.33	210.34 ± 7.30	209.29 ± 10.76
NO_2_ (µg/l)						
Inflow	42.55 ± 1.66	43.4 ± 1.08	71 ± 1.34	69.47 ± 1.33	57.4 ± 1.56	57.97 ± 1.66
Outflow	48.73 ± 2.01	42.67 ± 1.78	57.3 ± 1.11	58.45 ± 1.45	34.15 ± 1.37	33.42 ± 1.04
NO_3_ (µg/l)						
Inflow	778.44 ± 1.45	774.51 ± 8.32	711.69 ± 5.45	688.92 ± 3.45	554.14 ± 7.33	559.68 ± 6.75
Outflow	340.48 ± 3.34	370.45 ± 4.67	307.89 ± 5.11	320.21 ± 3.63	357.04 ± 5.67	345.61 ± 4.45
PO_4_ (µg/l)						
Inflow	51.43 ± 1.34	50.3 ± 2.26	72.08 ± 2.23	70.49 ± 1.08	78.24 ± 1.19	81.37 ± 1.28
Outflow	67.51 ± 2.12	45.4 ± 1.80	74.25 ± 3.03	72.68 ± 1.04	74.53 ± 2.00	72.96 ± 1.54
TP (µg/l)						
Inflow	152.63 ± 3.23	148.81 ± 2.23	142.86 ± 2.34	140.72 ± 2.23	135.71 ± 3.45	149.28 ± 2.67
Outflow	155.1 ± 3.45	140.8 ± 2.33	136.54 ± 2.93	140.15 ± 2.54	149.1 ± 3.76	147.46 ± 3.34

*HLR, hydraulic loading rate = water flow rate (Qw)/total surface area of pond or hydroponic trough; Qw = 16 m^3^ × 6 h; *EC* electrical conductivity, *DO* dissolved oxygen, *BOD* biological oxygen demand, *COD* chemical oxygen demand, *TA* total alkalinity, *NH*_*3*_ ammonia, *NO*_*2*_ nitrate, *NO*_*3*_ nitrite, *PO*_*4*_ orthophosphate, *TP* total phosphorus; ^#^total area by m, *IMTA* Integrated Multi-Trophic system, *NFT* Nutrient Film Technique, *FRS* Floating Raft Systems.

**Table 2:** Effect of hydraulic loading rates and water flow rate on the production of different studied vegetables and aquatic animals in IMTA systems in both first and second cycles.

Items	Experimental treatments					
	IMTA		IMTA-FRS		IMTA-NFT	
	First cycle	Second cycle	First cycle	Second cycle	First cycle	Second cycle
Total area (m)	40	40	20	20	15	15
Qw (m^3^ day-^−1^)	96	96	96	96	96	96
HLR (m day^−1^)	2.4	2.4	4.8	4.8	6.4	6.4
Vegetables (kg)						
Bell pepper	–	–	122.20	148.60	–	–
Chili pepper	–	–	105.87	99.40	–	–
Eggplant	–	–	127.40	118.23	–	–
Red leaf Lettuce	–	–	39.20	35.20	76.12	82.22
Green leaf Lettuce	–	–	38.80	37.60	73.68	89.54
Head lettuce	–	–	62.80	62.40	120.78	132.98
Tomato	–	–	210.97	256.68	–	–
Cucumbers	–	–	172.80	184.00	–	–
Broccoli	–	–	35.45	48.20	–	–
Aquatic animals (kg)						
Nile tilapia	121.99	157.12	–	–	–	–
Catfish	305.86	323.00	–	–	–	–
Mullets	65.21	119.69	–	–	–	–
Prawn	14.96	23.51	–	–	–	–
Mussels	248.60	245.52	–	–	–	–
Total, kg	756.62	868.83	915.49	990.31	270.58	304.74

*HLR, hydraulic loading rate = water flow rate (Qw)/total surface area of the pond or hydroponic trough; Qw = 16 m^3^ × 6 h; ^#^total area by m, *IMTA* Integrated Multi-Trophic system, *NFT* Nutrient Film Technique, *FRS* Floating Raft Systems.

### Production of experiments vegetable crops

Table 2 shows the production values for various tested vegetable species. Using 20 m^2^ of cucumber, broccoli, tomato, and eggplant-producing crops, the IMTA-FRS yielded 184.00, 48.20, 256.68, and 118.23 kg, respectively. The results showed that using 3 plants m^−1^ and 15 plants m^−2^ for head lettuce as a higher density in IMTA-NFT (122 m) and IMTA-FRS (20 m^2^) produced 132.98 and 62.40 kg, respectively. A total production of red and green leaf lettuce (82.22 and 89.54 plants) and (35.20 and 37.60 kg) was observed in IMTA-NFT and IMTA-FRS at densities of 3 and 15 plant m^−2^, respectively. In an IMTA-FRS of 20 m^2^ area, growing chili and bell pepper at a density of 8 plants m^−2^ yielded 99.40 and 148.60 kg, respectively (Table 2).

### Aquatic animal biomass production

The growth performance and feed utilization for different cultured aquatic animals are presented in Tables 3 and 4. Tilapia, *O. niloticus*, and catfish, *C. gariepinus* had final body weights of 275.65 g and 815.65 g, respectively. The feed intake (g/fish) for *O. niloticus* and *C. gariepinus* were 326.40 and 974.38 (g/fish/147 days), respectively, resulting in fish gain biomass of 255.25 and 684.38 g/fish. Uneaten feed, feces, and dissolved nutrients discharged from tilapia and catfish ponds are re-captured in the IMTA system by subsequent extractive aquatic species (e.g., mullet, prawns, and mussels) through feeding, swimming, and burrowing activities in the culture pond, where they serve as nourishment and acting as living filters. As a result, FCR, PER, PPV, and ER values (Table 4) are estimated for these species. The final body weights of mullets, prawns, and mussels in the IMTA system were 117.34, 25.89, and 487.14 g animal^−1^, respectively. Since mullets and prawns do not supply feed, thus freshwater mussels obtain nutrients by filtering microalgae and organic particulate matter from mullet and prawn pond discharge effluent.

**Table 3:** Growth performance parameters for different aquatic animal species used in the first and second cycles of the study.

Species	IBW (g/animal)		FBW(g/animal)		WG (g/animal)		SGR (% day)		S^#^	
	First cycle	Second cycle	First cycle	Second cycle	First cycle	Second cycle	First cycle	Second cycle	First cycle	Second cycle
Nile tilapia	28.30 ± 1.22	20.4 ± 1.70	221.00 ± 1.78^b^	275.65 ± 2.14^a^	192.7 ± 1.77^b^	255.25 ± 1.73^a^	1.40 ± 0.21^b^	1.77 ± 0.30^a^	92	95
Catfish	161.00 ± 26.40	131.27 ± 7.60	788.30 ± 5.10^b^	815.65 ± 8.20^a^	627.3 ± 5.11^b^	684.38 ± 8.10^a^	1.08 ± 0.31^b^	1.24 ± 0.22^a^	97	99
Mullets	3.20 ± 0.010	5.3 ± 0.01	85.80 ± 2.10^b^	117.34 ± 1.69^a^	82.60 ± 2.11^b^	112.04 ± 1.70^a^	2.24 ± 0.90^b^	2.11 ± 0.17^a^	38	51
Prawn	0.29 ± 0.010	0.20 ± 0.01	15.90 ± 1.70^b^	25.89 ± 0.56^a^	15.61 ± 1.70^b^	25.69 ± 0.61^a^	2.72 ± 0.71^b^	3.31 ± 0.32^a^	28	27
Mussels	214.46 ± 15.20	125.34 ± 13.40	486.5 ± 15.60^a^	487.14 ± 26.1^b^	272.04 ± 15.2^b^	361.8 ± 36.20^a^	0.59 ± 0.15^b^	0.92 ± 0.42^a^	67	66

^#^An estimate values, *IBW Initial body weight (g/animal), *FBW* Final body weight (g/animal), *WG* Weight gain (g/animal), *SGR* specific growth rate (%/day). The presented data are Mean ± SD (*n* = 3). The values followed by different lowercase letters are significantly different (*p* < 0.05).

**Table 4:** Feed utilization indices for different aquatic animal species used in the first and second cycles of the study^*^

Species	FI (g/animal)		FCR		PER		PPV		ER	
	First cycle	Second cycle	First cycle	Second cycle	First cycle	Second cycle	First cycle	Second cycle	First cycle	Second cycle
Nile tilapia	274.85	326.40	1.43 ± 0.11^b^	1.28 ± 0.11^a^	2.50 ± 0.10^a^	2.78 ± 0.22^b^	38.43 ± 1.40^a^	45.42 ± 1.64^b^	24.94 ± 1.87^b^	29.33 ± 1.67^a^
Catfish	976.83	974.38	1.56 ± 0.18^b^	1.42 ± 0.24^a^	2.29 ± 0.17^a^	2.50 ± 0.63^b^	40.16 ± 1.21^a^	43.92 ± 1.23^b^	24.37 ± 1.78^a^	27.53 ± 1.14^b^
Mullets	–	–	1.49^b§^*	1.24^a§^	2.57^a§^	2.88^b§^	43.49^a§^	49.72^b§^	27.01^a§^	31.42^b§^
Prawn	–	–	1.36^b§^*	1.21^a§^	2.61^a§^	2.95^b§^	43.98^a§^	50.73^b§^	27.27^a§^	31.90^b§^
Mussels	–	–	1.05^b§^*	0.90^a§^	3.39^a§^	3.94^b§^	47.54^a§^	56.45^b§^	28.87^a§^	34.60^b§^

^§^An estimate values, *FCR cumulative feed conversion ratio, *PER* Protein efficiency ratio (%), *PPV* Protein productive value (%), *ER* Energy retention (%). The presented data are Mean ± SD (*n* = 3). The values followed by different lowercase letters are significantly different (*p* < 0.05).

### Influences of IMTA system on feed utilization and feed conversion ratio

**Figure 2:**
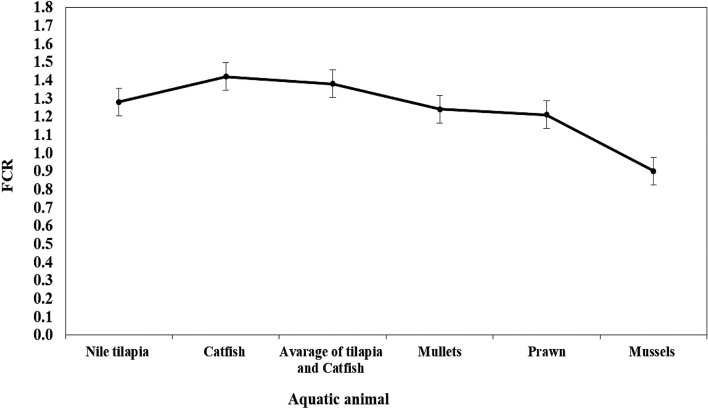
Cumulative values of estimated feed conversion ratios (FCR) obtained by different aquatic animal species to the IMTA-system.

The total biomass of all aquatic animals in the IMTA system was 736.46 kg, as opposed to 145.49 kg for tilapia and 271.01 kg for catfish in monoculture systems. Given that tilapia and catfish were the only two feeding species in the IMTA system, the FCRs for Nile tilapia and catfish as monoculture systems were 1.28 and 1.42, respectively. The apparent FCR IMTA-system improved as different extractive aquatic species were introduced. Mullets, prawns, and mussels had estimated cumulative FCR values of 1.24, 1.21, and 0.90 (Fig. 2). The same pattern may be observed with PER, PPV, and ER values (Table 4). Table 5 shows the proximal body composition of cultivated aquatic species. Catfish, tilapia, and mullets showed higher levels of body protein than prawns and mussels. The lowest level of whole-body lipid content in prawns and mussels was associated with the highest levels of body ash.

**Table 5:** Proximate body analysis of different aquatic animal species used in the first and second cycles of the study

Species	DM		CP		EE		ASH		GE (KJ\KG)	
	First cycle	Second cycle	First cycle	Second cycle	First cycle	Second cycle	First cycle	Second cycle	First cycle	Second cycle
Nile tilapia	26.04 ± 0.2	27.87 ± 1.20	59.13 ± 0.8	58.53 ± 1.19	28.16 ± 1.43	27.58 ± 1.43	12.71 ± 0.1	13.89 ± 2.18	600.20 ± 3.1	591.33 ± 5.38
Catfish	29.08 ± 0.4	29.08 ± 1.23	60.4 ± 0.2	60.40 ± 0.99	24.57 ± 0.92	26.57 ± 0.92	15.03 ± 0.7	13.03 ± 2.15	573.45 ± 4.2	573.45 ± 6.01
Mullets	29.66 ± 0.8	31.16 ± 1.32	53.29 ± 1.3	56.79 ± 1.43	27.87 ± 1.8 1	25.64 ± 1.8 1	18.84 ± 0.9	17.57 ± 2.35	564.46 ± 3.6	551.86 ± 8.54
Prawn	22.05 ± 1.1	27.92 ± 1.22	49.76 ± 0.9	51.45 ± 1.05	14.08 ± 1.2 4	9.52 ± 1.2 4	36.16 ± 1.9	39.03 ± 2.87	414.20 ± 5.1	408.91 ± 3.44
Mussels	12.17 ± 1.3	14.48 ± 1.54	37.76 ± 1.1	39.84 ± 2.34	5.54 ± 0.86	7.37 ± 0.86	56.70 ± 1.1	52.79 ± 1.55	265.70 ± 1.1	294.74 ± 9.49

*DM* dry matter, *CP* crude protein, *EE* ether extract, *GE* gross energy.

### Nitrogen (N) balance

**Figure 3:**
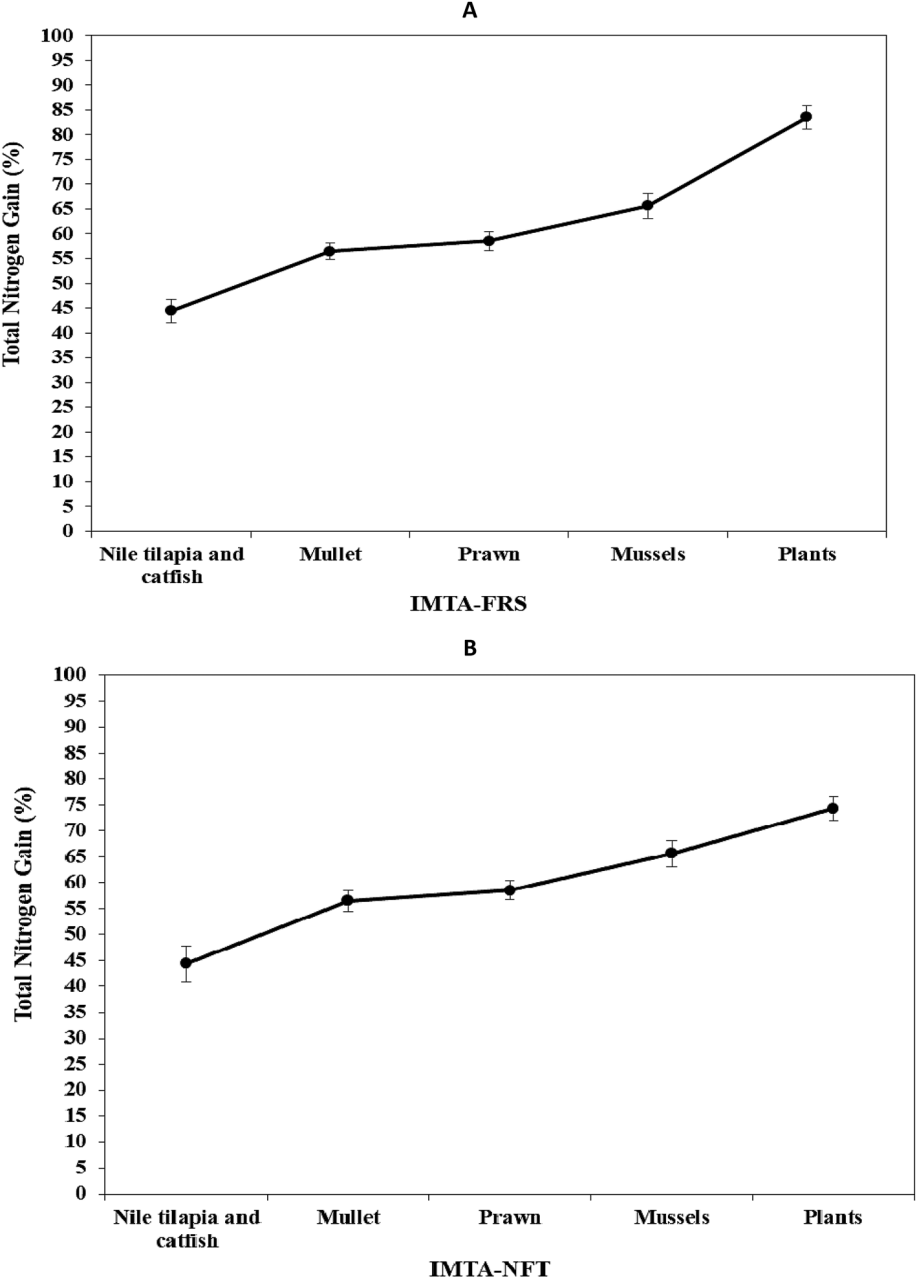
Cumulative enhancement of dietary nitrogen gain intake (%) obtained by different aquatic animal species and plants in the IMTA-FRS (**A**) and IMTA-NFT system (**B**).

In the IMTA system, tilapia and catfish consumed 186.05 and 385.85 kg of feed 40 m^−3^, containing 8.36 and 17.34 kg N, respectively, and gained 145.49 and 271.01 kg 40 m^−3^, containing 3.80 and 7.62 kg N, accounting for 45.40 and 43.91% of total dietary N supplied (Table 6). The remaining N was released in the dissolved excrement in water and discharged into mullet and prawn ponds. The retention efficiency of dietary N in mullet and prawn as detrivores aquatic animals was increased by 12.58 and 2.08%, respectively. The addition of mussels to the IMTA system boosted dietary N retention by 6.55%. Combining the IMTA with the FRS and/or NFT systems increases dietary N retention from 65.61 to 83.51% and 74.29%, respectively (Fig. 3).

**Table 6:** Total N biomass budgets for different aquatic animal species used in the first and second cycles of the IMTA-FRS and IMTA-NFT systems

Items*	Experimental treatments							
	IMTA-FRS				IMTA-NFT			
	kg-N/40 m^3^		% (N-gain/intake)		kg-N/40 m^3^		% (N-gain/intake)	
	First cycle	Second cycle	First cycle	Second cycle	First cycle	Second cycle	First cycle	Second cycle
Total N intake for tilapia	6.82	8.36	–	–	6.82	8.36	–	–
Total N intake for catfish	17.03	17.34	–	–	17.03	17.34	–	–
Total N intake for tilapia and catfish	23.85	25.70	–	–	23.85	25.70	–	–
Total N gain for tilapia	2.62	3.80	38.46	45.40	2.62	3.80	38.46	45.40
Total N gain for catfish	6.84	7.62	40.16	43.91	6.84	7.62	40.16	43.91
Total N gain for tilapia and catfish	9.46	11.41	39.67	44.40	9.46	11.41	39.67	44.40
Total N discharge from tilapia and catfish	14.39	14.29	60.33	55.60	14.39	14.29	60.33	55.60
Total N gain for mullet	1.59	3.23	6.66	12.58	1.59	3.23	6.66	12.58
Total N gain for prawn	0.25	0.54	1.07	2.08	0.25	0.54	1.07	2.08
Total N gain for mullet and prawn	1.84	3.77	7.73	14.66	1.84	3.77	7.73	14.66
Total N discharge from mullet and prawn	12.55	10.52	52.60	40.94	12.55	10.52	52.60	40.94
Total N gain for mussels	1.02	1.68	4.29	6.55	1.02	1.68	4.29	6.55
Total N discharge from mussels	11.52	8.84	48.32	34.39	11.52	8.84	48.32	34.39
Total N gain for plants in FRS	3.84	4.6	16.10	17.90	–	–	–	–
Total N gain for plants in NFT	–	–	–	–	1.26	2.23	5.28	8.68
Total N remaining from the system	7.68	4.24	32.22	16.49	10.26	6.61	43.03	25.71

*Nutrients (N or P) discharge (g) = Dietary nutrients (N or P) content (g) − fish body deposition nutrients (N or P) (g); Total Nutrient gain (g) = Tissue Nutrient gain (g) × total biomass production (g wet weight); Nutrient intake (g) = Total feed intake (g dry weight) × Nutrient feed content (%); Total Nutrient gain fish (g) = (Final body weight (g) × final tissue Nutrient content (%)) − (Initial body weight (g) × initial tissue Nutrient content (%)); Nutrient discharge (g) = Nutrient intake (g) − Nutrient fish gain (g wet weight); Nutrient (N or P) gain biomass (g) (mullets, prawn, mussels) = (Final body weight (g) × final tissue Nutrient content (%)) − (Initial body weight (g) × initial tissue Nutrient content (%)); Nutrient discharge (g) (mullets and prawn) = Nutrient discharge form Nile tilapia pond (g) + Nutrient discharge form catfish pond (g)) − Nutrient (N or P) gain biomass for mullets and prawn pond (g); Nutrient discharge form mussels (g) = Nutrient discharge form mullets and prawn pond (g) − Nutrient (N or P) gain biomass for mussels (g).

### Phosphors (P) balance

**Figure 4:**
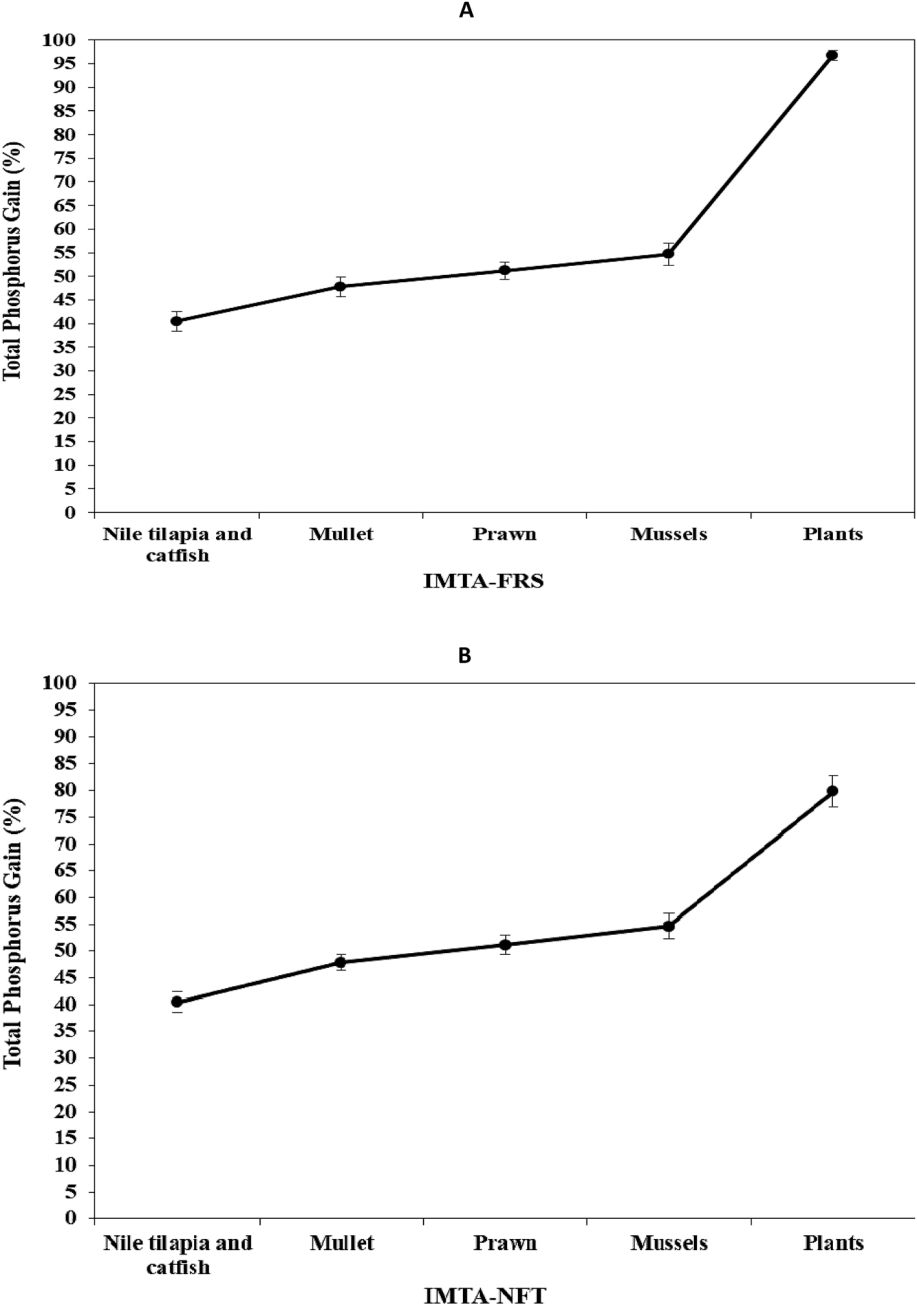
Cumulative enhancement of dietary phosphorus gain intake (%) obtained by different aquatic animal species and plants in the IMTA-FRS (**A**) and IMTA-NFT system (**B**).

The results showed that Nile tilapia and catfish consumed 186.05 and 385.85 kg of feed 40 m^−3^, including 2.44 and 7.27 kg of P, respectively. Tilapia and catfish gained 145.49 and 271.01 kg 40 m^3^, containing 1.30 and 2.62 kg of P, accounting for 53.47 and 36.10% of the total dietary P provided, respectively (Table 7). The remaining P feed was released as feces or dissolved in water and discharged into mullet and prawn ponds. Mullet and prawn, as detrivores aquatic animals, showed a 7.36 and 3.37% increase in dietary P retention, respectively. The addition of mussels to the IMTA system boosted dietary P retention by 3.48%. This finding shows that combining the IMTA with the FRS and/or NFT systems increases dietary N retention from 54.85 to 96.81% and 79.81%, respectively (Fig. 4).

**Table 7:** Total P biomass budgets for different aquatic animal species used in the first and second cycles of the IMTA-FRS and IMTA-NFT systems

Items*	Experimental treatments							
	IMTA-FRS				IMTA-NFT			
	kg-P/40 m^3^		% (P-gain/intake)		kg-P/40 m^3^		% (P-gain/intake)	
	First cycle	Second cycle	First cycle	Second cycle	First cycle	Second cycle	First cycle	Second cycle
Total P intake for tilapia	2.05	2.44	–	–	2.05	2.44	–	–
Total P intake for catfish	7.29	7.27	–	–	7.29	7.27	–	–
Total P intake for tilapia and catfish	9.34	9.70	–	–	9.34	9.70	–	–
Total P gain for tilapia	0.89	1.30	43.38	53.47	0.89	1.30	43.38	53.47
Total P gain for catfish	2.36	2.62	32.35	36.10	2.36	2.62	32.35	36.10
Total P gain for tilapia and catfish	3.25	3.93	34.77	40.46	3.25	3.93	34.77	40.46
Total P discharge from tilapia and catfish	6.09	5.78	65.23	59.54	6.09	5.78	65.23	59.54
Total P gain for mullet	0.46	0.71	4.97	7.36	0.46	0.71	4.97	7.36
Total P gain for prawn	0.24	0.33	2.62	3.37	0.24	0.33	2.62	3.37
Total P gain for mullet and prawn	0.71	1.04	7.59	10.73	0.71	1.04	7.59	10.73
Total P discharge from mullet and prawn	5.38	4.74	57.65	48.81	5.38	4.74	57.65	48.81
Total P gain for mussels	0.17	0.34	1.78	3.48	0.17	0.34	1.78	3.48
Total P discharge from mussels	5.22	4.40	55.86	45.33	5.22	4.40	55.86	45.33
Total P gain for plants in FRS	3.47	4.09	37.16	42.15	–	–	–	–
Total P gain for plants in NFT	–	–	–	–	1.37	2.44	14.67	25.14
Total P remaining from the system	1.75	0.31	18.70	3.19	3.85	1.96	41.19	20.19

*Nutrients (N or P) discharge (g) = Dietary nutrients (N or P) content (g) − fish body deposition nutrients (N or P) (g); Total Nutrient gain (g) = Tissue Nutrient gain (g) × total biomass production (g wet weight); Nutrient intake (g) = Total feed intake (g dry weight) × Nutrient feed content (%); Total Nutrient gain fish (g) = (Final body weight (g) × final tissue Nutrient content (%)) − (Initial body weight (g) × initial tissue Nutrient content (%)); Nutrient discharge (g) = Nutrient intake (g) − Nutrient fish gain (g wet weight); Nutrient (N or P) gain biomass (g) (mullets, prawn, mussels) = (Final body weight (g) × final tissue Nutrient content (%)) − (Initial body weight (g) × initial tissue Nutrient content (%)); Nutrient discharge (g) (mullets and prawn) = Nutrient discharge form Nile tilapia pond (g) + Nutrient discharge form catfish pond (g)) − Nutrient (N or P) gain biomass for mullets and prawn pond (g); Nutrient discharge form mussels (g) = Nutrient discharge form mullets and prawn pond (g) − Nutrient (N or P) gain biomass for mussels (g).

## Discussion

Aquaculture is vital for food security, employment, and economic development in the face of climate change and population expansion. IMTA systems capture and recycle fish waste, decreasing concentrations in waters and, consequently environmental harm^28^. Aquaponics-based fish pond drainage that is recovered by growing vegetable crops and recycled back into fish ponds may increase the sustainability of aquaculture even more. As a result, the current study makes a substantial contribution to the field of IMTA-aquaponics science by providing novel data on freshwater IMTA water usage and the potential for wastewater reductions associated with aquaponics varied vegetable crop production^29^.

The IMTA system can be used for freshwater, brackish water, marine, open water, and land-based aquaculture in temperate to tropical regions^19^. To better optimize nutrient flow and uptake, this study used an integrated multi-nutrient aquaculture (IMTA) system for land-based culture of Nile tilapia, Nile catfish, gray thin mullet, freshwater prawns, and freshwater oysters in a modular system. Aquaponics is a form of freshwater IMTA production^30^ because it involves at least two species, such as plants and fish. The two species utilize nutrient sources and play distinct functions in aquatic ecosystems^31^. Several freshwater IMTA studies^20,29,32–38^ show that the IMTA system may improve agricultural environmental conditions while also increasing overall biomass production through product diversity.

Applying IMTA to marine environments is an agricultural model for commercial and pollution control. The marine IMTA system uses commercial and environmentally beneficial species^39^. The results of many marine waters IMTA^40–46^ indicated that deploying the IMTA system to marine waters enhances water quality in culture medium while also increasing system profitability and balance. Further research should be undertaken on a wider scale with a variety of potential local extractive species to better understand system performance, the proportion of species used in systems, and the integration of IMTA with other systems such as Biofloc Technology and Recirculating Aquaculture Systems.

To bring environmental sustainability to aquaculture systems, in situ removal/utilization of inorganic and organic nutrients could be achieved biologically. IMTA involves introducing un-fed aquatic animals such as mullet and prawn, as well as organic extractive species such as mussels^47^. This system efficiently utilizes organic and particulate waste. Plants grown in an IMTA-aquaponic system are recognized as one of the most effective ways to use inorganic nutrients such as nitrogen and phosphorus^48^. As a result, the current IMTA-Aquaponic model was found to be both productive and economically viable, with an environmental in-situ bio-remediation impact.

In the current study, controlling the temperature in an aquaponic system poses a cost barrier in low-horticulture technology. The only choices for adjusting temperature in hot summers and cold winters are a shade cloth and a plastic sheet over the crops and fish-pond greenhouses. However, one of the most difficult jobs completed throughout this investigation was to keep the water temperature nearly constant while also ensuring that the values were adequate for all different cultured organisms and plants. The water temperature in the fish ponds is maintained between 24 and 28 °C depending on the season. Since higher water temperatures may restrict fish and plant growth. However, during the summer months, the temperature within the greenhouse will rise significantly, which can be stressful for certain plants and increase the rate of water evaporation from the system. However, installing the system in a frame that can be shaded in the summer to keep the interior of the greenhouse cooler and covered with plastic in the winter to keep the interior of the greenhouse hotter would be the best method. In contrast to IMTA-NFT, all experimental plants grew positively in the IMTA-FRS hydroponic trough with no symptoms of nutritional or mineral imbalance. The production of lettuce species increased as the hydraulic loading rate increased up to 4.8 m day^−1^ in IMTA-FRS and to 6.4 m day^−1^ in IMTA-NFT. However, the yield did not differ much between the first and second cycles. The continuous water flow operation of the various aquaponics systems demonstrated that when HLR increased, so did the % removal of BOD, TSS, TAN, and Nitrite-N. Unlike BOD, TSS, and Nitrite-N, the removal % of Nitrate–N and TP increased with HLR from 0.64 to 1.28 m day^−1^ and declined with HLR from 1.28 to 4.8 m day^−1^. TSS, BOD, TAN, Nitrite-N, Nitrate–N and TP values in water effluent are consistent with previous studies^49,50^.

Studies by Ramírez‐Rochín et al.^51^, Valencia-Castañeda et al.^52^, and Alarcón-Silvas et al.^53^, have found that increasing NO_3_-N leads to improved TAN elimination, indicating greater nitrification. Since NO_3_-N accumulates in the system, once NH_3_-N is nitrified, denitrification becomes limited. Inadequate residence/retention times for the biofilter sump to denitrify NO_3_-N, the presence of DO, and a lack of available carbon in the system are all potential concerns that could impede denitrification. There are several ways to remove NO_3_-N from wastewater. One potential method of elimination is for plants to absorb NO_3_-N from growing media via the root zone. Microbial assimilation is a supplementary approach for particle removal. It can also be absorbed by bacteria in the water column or biofilms on plant root mats^54^.

The pH stability is essential in aquaponics since it is required by all living species in a cycling system, including fish, plants, and microbes^55^. Each living component has a distinct optimum pH. Most plants require a pH of 6–6.5 to optimum nutrient uptake. Aquatic animals are those that thrive in pH levels ranging from 7.0 to 9.0. Nitrifying bacteria have an optimal pH of more than 7. The value of pH during the tested period was adequate to ensure normal activity for nitrifying bacteria from the biological filter unit. During this study, the pH was kept near 7. When the pH in the sedimentation pond rose over 8.0, the freshwater well was manually fed into the system (via the fish ponds) to lower the pH to 7.0, bringing it down to the optimal water pH.

The type of fish used in an aquaponics system is determined by the climate of the culture environment and, as well as the optimal temperature that the grower can maintain for maximum growth. Several warm-water and cold-water fish species have adapted to IMTA-aquaponics systems, including tilapia, trout, *Oncorhynchus mykiss*, perch, *Perca fluviatilis*, Arctic char, *Salvelinus alpinus*, and seabass, *Dicentrarchus labrax*. However, tilapia and catfish are raised in the majority of commercial aquaponics systems because they are fast-growing, resilient, and tolerant of crowding and generally poor water quality conditions, including being extremely tolerant to variations in dissolved oxygen levels, temperature, pH, and dissolved oxygen, solids, as well as resistance to a variety of illnesses that affect other farmed fish. Furthermore, tilapia and catfish can tolerate temperatures ranging from 18 to 35 °C and can grow successfully at temperatures above 22 °C^56^, covering the temperature range required for plant production. Tilapia and catfish are both popular in restaurants and markets because their meat may be used for both domestic and wholesale purposes.

Prawn-mullet polyculture has been proposed as a way to create a sustainable aquaculture system. In a polyculture system setup, fish and prawns can occupy a variety of cultural niches. In a large extensive farm, fish can filter phytoplankton and zooplankton from the upper water column. The prawn spends the majority of its time on the pond bottom, feeding on bacterial films on the substrate and detritus that settles from above. In a more intense farm, fish monopolize the pelleted; however, certain feed particles constantly sink to the bottom, are obtained by the prawn. Furthermore, in the current study, tilapia and catfish feces contribute to the detritus source of nutrition that sustains the prawn-mullet polyculture, increasing overall fish and prawn yield^57^. According to Dickson et al.^58^, aquatic polyculture has been identified as a viable strategy for boosting farm cost-effectiveness since it can lower average production costs, increase system yields, and reduce economic risks associated with monoculture operations. Polyculture also promotes ecological stability and recycling processes, which can lead to synergistic benefits for participating species. This aquaculture synergy can boost profitability by accelerating growth rates and/or reducing feed supply.

The growth induces and feed utilization values for different aquatic animal cultures in the testing IMTA system showed that Nile tilapia, catfish, mullets, prawns, and freshwater mussels are highly adaptable and resilient to environmental conditions. Otazua et al.^59^ has previously demonstrated that IMTA-aquaponics systems outperform aquatic monoculture systems. In the present study, about 55.60% and 59.54% of the dietary N and P intakes of tilapia and catfish, respectively, are lost as feces, pseudo-feces, and uneaten feed that sink to the pond bottom in a monoculture system. In the current study, approximately 55.60% and 59.54% of tilapia and catfish dietary N and P intakes, respectively, are discharged as feces, pseudo-feces, and uneaten feed that sink to the pond bottom in a monoculture system.

The addition of diverse extractive aquatic species to the IMTA-system increased the utilization efficiency of dietary N and P intake while also supplying more biomass to the system without the need for further feed. Coupled IMTA systems, either FRS or NFT, increase the nutrient retention efficiency of dietary N and P by 83.51% and 96.82%, respectively. This study shows that an IMTA-aquaponic system can alleviate negative circumstances and external environmental consequences in a monoculture system.

Suspended feeders, such as mussels and bivalves, improve water quality by filtering suspended particles and producing feces and pseudo-feces^60^, both of which reduce turbidity in water^61^. Mussel clearance rates are frequently associated with water flow rates, food quantity, and water temperature^62^. Mussels retained 6.55% and 3.48% of their dietary N and P intakes, respectively. There is no information available on the culture of freshwater mussels. The effects of unionid mussels on impacts on N and P retention are rarely examined^63^. Overall, introducing unionid freshwater mussels into bodies of water may boost P retention in soft tissues and shells while reducing N loss via denitrification. Ecological managers may find more support for unionid population restoration if the ecological role of unionids in nutrient retention and removal is well understood.

The results showed that the apparent IMTA-FCR was 0.90, which is better than the industry standard FCR of 1.50 for intensively commercial tilapia or catfish monoculture systems^54^. As a result, the IMTA system appears to be one of the most effective ways to boost feed utilization in aquaculture. Feed utilization efficiency induces such as PER, PPR, and ER followed the same pattern. The majority of the previous studies found that when combined with a monoculture fish production system, aquaponics had no significant effect on FCR, WG, or SGR values in tilapia or catfish. Combining *O. niloticus* with two different plants, *Phaseolus vulgaris,* and *Brassica rapa chinensis*^64^ achieved a comparable result in aquaponic cultivation. In terms of productivity, product diversification, co-cultural benefits, and FCR, polyculture outperforms monoculture.

Tian et al.^65^ found that fish polyculture improves pond water and sediment quality while lowering waste emissions^66^. All polyculture combinations were superior to monoculture in terms of economic and ecological efficiency^67^. One possible explanation is that IAAS has been recommended as one of several farming systems capable of mitigating some of the environmental issues associated with monoculture while improving total productivity in a given site^68^.

Many plant crops can be cultivated in aquaponics systems, although some are more suited than others. When deciding which crop to plant, the grower's goal should come first and foremost. Crops having a high market value and a short harvesting time will be more suitable if the venture's purpose is to generate a profit, as is the case with commercial-scale systems. The protocol for the trials in the current investigation was amended based on the data collected during the first production cycle. Plant selection, for example, was amenable to change when any sort of plant (for example, lettuce species, peppers, tomato, cucumber, and broccoli…etc.) performed poorly under the experimental conditions. Lettuce is the most popular aquaponic crop owing to its short harvesting period (3–4 weeks) and high demand in diets^69^; it is also a very profitable crop since a considerable amount of its final mass is harvestable and has high market acceptance. Another reason these crops thrive is the lack of a fruiting stage, which keeps nutritional requirements stable and results in a more consistent harvest. According to the study's findings, broccoli, tomato, cucumber, and eggplant acclimated quickly to the aquaponics environment in IMTA-FRS systems but not in IMTA-NFT systems, but lettuce species adapted well to the aquaponics environment in all experimental conditions. This is because the density of fish culture stocking influences the selection of plant species suited for aquaponic systems, as well as, the nutritional content of aquaculture effluent. Lettuce has low to medium nutritional requirements, making it ideal for IMTA-NFT aquaponic systems, which require less water than IMTA-FRS systems. Fruit-bearing plants (tomatoes, peppers, broccoli, and cucumbers) require more nutrients and perform better in IMTA-FRS systems which use more water than IMTA-NFT systems.

The hydroponic unit is designed to serve two purposes. First, the unit must allow water to flow over the plant roots so that the plant may take necessary nutrients. Second, the unit must mechanically support the plants. The amount of water that should flow to the hydroponic unit varies depending on the unit size, as well as the number and size of the plants being cultivated^70^. In the present study, two hand valves were installed in line ahead of the hydroponic unit with the fish culture pond to allow manual control of water flow to each. Typically, the bypass water line returns the majority of the pump's output to the fish culture pond. An aeration nozzle attached to the line's output helps maintain dissolved oxygen levels safe (> 5 ppm, 5 mg/l) in different culture ponds. In a backyard aquaponics system, the water flow rate (Qw) was determined as the volume of water equivalent to the fish tank volume passing through the biofilter twice per hour. The current design has a water flow rate of 16 m^3^ h (× 6 h). Increasing water flow rates improves the removal of BOD, TSS, NH_3_-N, and NO_2_-N, in the IMTA-NFT and IMTA-FRS systems.

Scaling up freshwater IMTA-Aquaponic will provide major advantages to farmers in similar topographic areas, particularly in Egypt. The combination of aquaculture and hydroponics expands farmers' income streams by allowing them to produce both fish and vegetables. This technique boosts farmers' profits and allows for a wider range of crop output. On the other hand, this technology allows farmers to recycle and use nutrients on-site, which reduces the need for chemical fertilizers. This approach is particularly useful for small-scale farms and areas with limited access to chemical fertilizers, as it enables farmers to enhance their nutrient management practices in situ. However, by employing this revolutionary approach, farmers may significantly decrease consumption of water compared to ordinary agricultural practices, making it especially valuable in water-scarce locations such as Egypt. Finally, this technology helps to reduce the environmental impact of aquaculture, which contributes to better ecosystem health. IMTA implementation is still underway in many countries. There are numerous aspects to consider while designing a freshwater IMTA system, such as increasing land use efficiency, because IMTA is a complex system and relies on natural energy use to be more energy efficient. Capital investment, maintenance, harvesting methods, and system design continue to be challenges and obstacles. The selection of appropriate species, as well as nutrient flow control, are crucial for enhancing output and creating optimal specimens that are well-suited to animal and plant growth circumstances. Future study needs to focus on improving the larger-scale system's design and operation, investigating the possibility of implementation, and undertaking economic evaluation to establish its practicality and profitability in diverse countries and environments.

As an innovative sustainable food production system, IMTA-aquaponics aims to maximize nutrient input while minimizing waste and achieving a zero-discharge recirculating system. More research on the solubility of fish waste in the IMTA-aquaponics system is required to convert all available nutrients into plant biomass. The goal is to determine the best fish feed composition for aquaponics so that the water nutrients content meets hydroponic requirements as nearly as feasible. As a result, we must determine the macronutrient and micronutrient proportions that fish in a given system can discharge into the water for a specific diet; these proportions differ based on fish species, fish density, temperature, and plant type.

## Conclusions

Aquaponics systems purify water by absorbing N and P from fish ponds and using it as fertilizer for plant growth, so recycling it rather than discarding it and harming the environment. The current experiment tested three different IMTA-aquaponics systems containing Nile tilapia, Nile catfish, thin-lipped grey mullet, freshwater prawns, and mussels with vegetables using a renewable energy system. Combining tilapia, catfish, mullets, prawns, and mussels boosted dietary N efficiency from 44.40 to 65.61%. Finally, adding a hydroponic system increased dietary N efficiency to 74.29 and 83.51%, respectively, for IMTA-NFT and IMTA-FRS. The IMTA system boosted dietary P efficiency from 40.46 to 54.67%, while adding a hydroponic system increased dietary P efficiency to 79.81 and 96.81%, respectively, for IMTA-NFT and IMTA-FRS. This finding implies that, in the face of global population growth, climate change, and declining water supplies in arable land, developing efficient and integrated aquaculture agriculture techniques like IMTA-Aquaponics systems will promote economic development while also providing a small-scale business opportunity for developing-country youth.

## Data Availability

Not applicable (this manuscript does not report data generation or analysis).
