# Author Correction: Optimizing nutrient utilization, hydraulic loading rate, and feed conversion ratios through freshwater IMTA-aquaponic and hydroponic systems as an environmentally sustainable aquaculture concept

**DOI:** 10.1038/s41598-024-70935-0

**Published:** 2024-09-03

**Authors:** Ashraf M. A.-S. Goda, Ahmed M. Aboseif, Mostafa K. S. Taha, Eman Y. Mohammady, Nevine M. Aboushabana, Hani M. Nazmi, Marwa M. Zaher, Hadir A. Aly, Mohamed A. S. El-Okaby, Nora Ibáñez Otazua, Mohamed Ashour

**Affiliations:** 1 National Institute of Oceanography and Fisheries, (NIOF), Cairo, Egypt; 2 INKOA SISTEMAS, S.L., Ribera de Axpe 11, Edificio D1, Dpto 208, 48950 Erandio, Spain

Correction to: *Scientific Reports* 10.1038/s41598-024-63919-7, published online 27 June 2024

The original version of this Article contained an error in Table 1, where the columns “IMTA”, “IMTA-FRS”, and “IMTA-NFT” where incorrectly aligned with the rows “First cycle” and “Second cycle”.

The original Article has been corrected.

